# Interactions between endoparasites and anurans in the Nhecolândia Pantanal, Brazil

**DOI:** 10.1590/S1984-29612025034

**Published:** 2025-06-30

**Authors:** Priscilla Soares dos Santos, Isabela Caroline Oliveira da Silva, Maiara Cabrera Miguel, Rodney Murillo Couto, Luiz Eduardo Roland Tavares, Vanda Lúcia Ferreira, Fernando Paiva

**Affiliations:** 1 Laboratório de Parasitologia Animal, Instituto de Biociências, Universidade Federal de Mato Grosso do Sul – UFMS, Campo Grande, MS, Brasil; 2 Programa de Pós-Graduação em Biotecnologia, Universidade Católica Dom Bosco – UCDB, Campo Grande, MS, Brasil; 3 Programa de Pós-Graduação em Biologia Animal, Universidade Federal de Mato Grosso do Sul – UFMS, Campo Grande, MS, Brasil; 4 Programa de Pós-Graduação em Ciência e Tecnologia Animal, Universidade Estadual Paulista “Júlio de Mesquita Filho” – UNESP, Ilha Solteira, SP, Brasil; 5 Laboratório de Pesquisa em Herpetologia, Instituto de Biociências, Universidade Federal de Mato Grosso do Sul – UFMS, Campo Grande, MS, Brasil

**Keywords:** Helminths, aquatic habitats, ecological networks, parasitism prevalence, Helmintos, habitats aquáticos, redes ecológicas, prevalência de parasitismo

## Abstract

We analyzed the distribution of endoparasites in five species of Hylidae frogs during their reproductive period, aiming to understand how the host's habitat influences the parasites. Five anuran species (*Boana raniceps*, *Pithecopus azureus*, *Pseudis platensis*, *Scinax acuminatus* and *Scinax nasicus*) were sampled in temporary and permanent ponds in Nhecolândia Pantanal, Brazil. A total of 276 individuals were collected, 43.84% of which were parasitized by endoparasites. Metacercariae of Diplostomidae gen. sp. were predominant, accounting for 85.94% of the total parasites found. Nematodes and cestodes were also identified, with nematodes representing 7.85% of the total parasites. Statistical analyses indicated that host body size was not a significant predictor of parasitism probability, although a weak negative correlation between host body size and parasite abundance was observed. Network analysis revealed a modular structure in the parasite-host interactions, with moderate connectivity. These results suggest that environmental factors, particularly those related to the aquatic habitats of anurans, play a fundamental role in shaping parasite community structure. This study provides important insights into the complex ecological interactions between anurans and their parasites, significantly contributing to the understanding of parasite-host interaction dynamics in the Pantanal ecosystem.

## Resumo

Foi analisada a distribuição de endoparasitas em cinco espécies de anuros da família Hylidae, durante seu período reprodutivo, buscando compreender como o habitat do hospedeiro influencia os parasitos. Cinco espécies de anuros (*Boana raniceps*, *Pithecopus azureus*, *Pseudis platensis*, *Scinax acuminatus* e *Scinax nasicus*) foram amostradas em lagoas temporárias e permanentes no Pantanal da Nhecolândia, Brasil. No total, 276 indivíduos foram coletados, dos quais 43, 84% estavam parasitados por endoparasitas. Metacercárias de Diplostomidae gen. sp. foram predominantes, correspondendo a 85,94% do total de parasitos encontrados. Também foram identificados nematódeos e cestódeos, com os primeiros representando 7,85% do total de parasitos. As análises estatísticas indicaram que o comprimento corporal do hospedeiro não foi um preditor significativo da probabilidade de parasitismo, embora uma fraca correlação negativa entre o comprimento e a abundância de parasitos tenha sido observada. A análise de redes evidenciou uma estrutura modular nas interações parasito-hospedeiro, com conectividade moderada. Os resultados sugerem que fatores ambientais, especialmente aqueles relacionados aos habitats aquáticos dos anuros, desempenham um papel fundamental na organização das comunidades de parasitas. Este estudo fornece informações importantes sobre as complexas interações ecológicas entre anuros e parasitas, contribuindo significativamente para a compreensão da dinâmica na interação parasito-hospedeiro no ecossistema do Pantanal.

## Introduction

Amphibians play a significant ecological role and can provide essential insights into the understanding of global biodiversity (Whittaker et al., 2013). The habitat conditions where these species are found have been the subject of considerable investigation (Pounds et al., 2006; Siqueira & Rocha, 2013; Mitchell & Bergmann, 2016; Sawada et al., 2022).

Hylidae (Amphibia: Anura) is widely recognized as one of the most species-rich anuran families (Faivovich et al., 2005). Although adults are primarily arboreal, they may be associated with various microhabitats and lifestyle categories (aquatic, semiaquatic, scansorial, fossorial, terrestrial, or riparian). These attributes contribute to this family’s broad variation in habitat use and, consequently, more diverse associations (Wells, 2007). In Neotropical regions, these anurans can occupy a variety of habitats, including open areas (Brasileiro et al., 2005), veredas (Sugai et al., 2014), forests, gallery forests, forest fragments, and edges (Gordo & Campos, 2003; Brasileiro et al., 2005; Juncá, 2006; Bastazini et al., 2007; Machado & Maltchik, 2007; Uetanabaro et al., 2007; Brasileiro et al., 2008; Pansonato et al., 2011; Prudente et al., 2013; Santos et al., 2014; Souza et al., 2017; Mônico et al., 2017; Dorigo et al., 2018).

In the Pantanal ecosystem, in Brazil, a floodplain ecosystem, the distribution patterns of anurans during the reproductive period are recognized for being influenced by the complexity and diversity of habitats present in the breeding sites (Prado et al., 2005). Moreover, annual flood pulses may facilitate the massive dispersal of species and enable the colonization of new environments (Delatorre et al., 2015). Understanding these patterns requires a critical appreciation of the interactions between anurans and the various microhabitats they utilize. Some examples of Hylidae species occupying microhabitats associated with water bodies in floodplains, mostly temporary ponds, as reported by Pansonato et al. (2011) and Delatorre et al. (2015), include *Boana raniceps* (Cope 1862), *Dendropsophus elianeae* (Napoli and Caramaschi, 2000), *D. nanus* (Boulenger, 1889), *Pithecopus azureus* (Cope, 1862), and *Scinax fuscomarginatus* (Lutz, 1925) in pasture areas; *B. raniceps*, *Scinax nasicus* (Cope, 1862), and *Trachycephalus typhonius* (Linnaeus, 1758) in forested areas; *D. elianeae*, *Dendropsophus melanargyreus* (Cope, 1887), *Dendropsophus minutus* (Peters, 1872), *Dendropsophus nanus*, *Lysapsus limellum* (Cope, 1882), *Pseudis platensis* (Galardo, 1961), *Scinax acuminatus* (Cope, 1882), *Scinax fuscovarius* (Lutz, 1925), *S. nasicus*, and *T. typhonius* in natural depressions; *S. acuminatus*, *S. nasicus*, and *T. typhonius* in peri-anthropic areas; *D. elianeae*, *D. melanargyreus*, *L. limellum*, *P. platensis*, and *S. fuscovarius* in artificial reservoirs; and *L. limellum* and *P. platensis* in ponds. Given this multiplicity of habitats occupied by hylid frogs during their intrinsic life history stages, it is plausible that such diversity provides opportunities for exposure to and interactions with various biotic groups, including different forms of endoparasites.

Since Darwin (1859), the struggle for existence has been identified as the inevitable result of the rapid multiplication of all organized beings. Parasites are defined by their ability to reach their hosts for survival and reproduction (Sasal & Thomas, 2005). In this context, the necessity of parasites is defined as an evolutionary strategy to ensure their survival amidst the constantly changing adaptations of their hosts (Van Valen, 1973). When considering the ways in which organisms survive, it is essential to understand the ecological niche (Hutchinson, 1957), which is defined as a multidimensional hypervolume composed by environmental variables (biotic and abiotic) in which a species can exist. Along this line of reasoning, Rohde (1979) emphasized that although the number of niche dimensions is almost infinite, some dimensions are sufficient to characterize the niche volume of a parasite with a high degree of precision. Additionally, these dimensions may include host specificity, geographic range, host sex and age, seasonality, diet, hyperparasitism, and the host's microhabitats and macrohabitats (Rohde, 1979).

The generality of parasites, measured by the exploitation of various host species, provides a framework that predicts how species respond to parasites in terms of behavioral traits. It also shows how temporal and spatial heterogeneity in host species' associations with parasites favors the evolution of adaptive antiparasitic behaviors (Van Buskirk, 2002; Koprivnikar et al., 2006; Koprivnikar & Randhawa, 2013; Marino Jr., 2016). This complex relationship, or interaction network, is fundamental to understanding ecological systems and their significance in the environment.

Among the types of interaction networks, parasite-host interactions, along with predator-prey interactions, are classified as antagonistic networks, which tend to be organized into internally connected compartments (or modules) with limited connections between them (Olesen et al., 2007; Thébault & Fontaine, 2010). These compartments may be related to selective forces as coevolutionary units concerning past evolutionary history and the high specificity of the groups (Thébault & Fontaine, 2010). According to Godfrey et al. (2013), parasite-host antagonistic networks can also be shaped by the host's use of the environment, as spatial heterogeneity can influence parasite infections.

For anuran amphibians, the structural heterogeneity of their habitat is a determining factor for their occupation of temporary and/or permanent ponds and diverse vertical strata (Campos et al., 2013; Haddad et al., 2013; Delatorre et al., 2015), exposing them to potential infections (Poulin & Morand, 2004; Hamann et al., 2009) resulting from interactions between tolerances and requirements that define the conditions and resources necessary for individuals and/or species to survive (Hutchinson, 1957). Habitat selection strongly influences the abundance and richness of parasites (Byrnes & Rohde, 1992; Rohde et al., 1995); thus, the host's use of the environment contributes to the establishment of different parasitic agents and/or infections according to the biological cycles of each parasite group (Lafferty, 2008).

Thus, considering the importance of parasite distribution in relation to the habitat of anurans, the scope of this study was to verify the distribution of endoparasites in five species of Hylidae that share the same reproductive habitat. In doing so, this study aims to elucidate the complex ecological interactions that shape the communities of the studied Hylidae and their respective endoparasites.

## Material and Methods

Five species of Hylidae anurans (*B. raniceps*, *Pithecopus azureus*, *Pseudis platensis*, *S. acuminatus*, and *S. nasicus*) were captured through nocturnal active searches during the reproductive period. Different temporary and permanent ponds (nonrepeating) were sampled in the Pantanal ecosystem, more specifically in the Nhecolândia subregion (18º59'S, 56º39'W), Corumbá municipality, Mato Grosso do Sul, west-central Brazil. For a detailed description of the study area, see Delatorre et al. (2015) and Nunes et al. (2021). Five expeditions were conducted, with an average sampling effort of four nights per campaign, during December 2014, March 2015, April and November 2016, and November 2017. The active search for anurans covered various microhabitats, from shrub vegetation and pond margin to the central portion of the pond, in the water and associated vegetation.

In the laboratory, the host body size was measured from the snout-vent length using a caliper with a precision of 0.02 mm (± 0.025 mm per 300 mm) in the metric system; and after painlessly killing the frogs using topical 5% lidocaine gel, necropsy was performed, and the body cavities and internal organs were inspected under a stereomicroscope for the collection of metazoan endoparasites. The parasite samples were separated, cleaned, and fixed according to Amato et al. (1991).

Morphological and morphometric analyses of the parasites were performed using a light microscope with phase contrast and a Leica DM5500™ model and a Leica M205™ model stereomicroscope equipped with a computerized system for image capture and processing (LAS 3.9™ Leica). For the taxonomic identification of the parasites found, the criteria proposed by Yamaguti (1961; 1971), Travassos et al. (1969), Vicente et al. (1990), and other taxonomic review articles were used. The voucher samples of the anurans and parasites were deposited in the Zoological Collection of the Federal University of Mato Grosso do Sul, Institute of Biosciences, Campo Grande, Mato Grosso do Sul.

The host species include *Pithecopus azureu*s (Cope, 1862) (ZUFMS-AMP12162-12237), *Boana raniceps* (Cope, 1862) (ZUFMS-AMP12238-12303), *Pseudis platensis* Gallardo, 1961 (ZUFMS-AMP12304-12364), *Scinax nasicus* (Cope, 1862) (ZUFMS-AMP12365-12422), and *Scinax acuminatus* (Cope, 1862) (ZUFMS-AMP1242-12437). The deposited endoparasites include *Brevimulticaecum* sp. (ZUFMS-NEM00067), *Oxyascaris oxyascaris* Travassos, 1920 (ZUFMS-NEM00070), *Raillietnema minor* Freitas and Dobbin Junior, 1961 (ZUFMS-NEM00071), Cosmocercidae gen. sp. (ZUFMS- NEM00073), *Cosmocerca parva* Travassos, 1925 (ZUFMS-NEM00068), *Cosmocercella minor* Steiner, 1924 (ZUFMS-NEM00069), *Aplectana hylambatis* Baylis, 1927 (ZUFMS-NEM00066), *Physaloptera* sp. (ZUFMS- NEM00072), *Rhabdias breviensis* Nascimento et al., 2013 (ZUFMS-NEM00074), *Choledocystus elegans* Travassos, 1926 (ZUFMS-PLA00035), *Catadiscus cohni* Travassos, 1926 (ZUFMS-PLA00036), *Catadiscus uruguayensis* Freitas and Lent, 1938 (ZUFMS-PLA00037), *Brachylaima* sp. (ZUFMS-PLA00038), *Rauschiella* sp. (ZUFMS-PLA00039), *Glypthelmins* sp. (ZUFMS-PLA00041), *Glypthelmins quieta* Stafford, 1905 (ZUFMS-PLA00042), and *Dero lutzi* Michaelsen, 1926 (ZUFMS- ANN00001).

The ecological descriptors of parasitism, including infection incidence, intensity, mean intensity, and mean abundance, were estimated according to Bush et al. (1997).

Additionally, the discrepancy index was calculated. Prevalence represents the proportion of hosts infected with one or more individuals of a specific parasite species (or taxonomic group) divided by the total number of hosts examined. When used descriptively, it is often expressed as a percentage, and when incorporated into mathematical models, it is expressed as a proportion. The mean intensity is obtained by the ratio of the total number of parasites of a particular taxon found in a sample to the number of hosts infected by that parasite. The mean abundance refers to the average number of individuals of a particular parasite species in a sample of a host species. This average is calculated using the total number of hosts examined, both infected and uninfected. The discrepancy index in parasitology is a statistical measure that reflects the variation in the distribution of parasites within a host population (Poulin, 1993). It is used to analyze whether parasitic infestations are uniformly distributed among hosts or if there is a significant discrepancy, indicating that some hosts carry a much larger parasitic load than others do. The discrepancy index ranges from d = 0 to 1. When d = 0, it indicates the absence of aggregation, meaning individuals are distributed randomly or uniformly within the community, with no concentration. On the other hand, when d = 1, it represents the theoretical maximum aggregation, meaning all individuals are concentrated in a single area or are highly clustered with no dispersion between them (Poulin, 1993).

Considering that each individual of a given anuran species represents a sampling unit for endoparasites, we applied diversity indices (Shannon-Wiener), Simpson's dominance (*D*), and Pielou's evenness (J) to assess the parasitic component community at the host species level. The structure of the parasite community was not assessed for *S. acuminatus* due to its low parasite abundance, which limited the analysis of the diversity and distribution of the parasitic community in this species.

Additionally, a logistic regression model was used to investigate the relationship between host body size and the probability of being parasitized, with the analysis conducted for all species combined. The null hypothesis (H_0_) was that there is no significant relationship between host body size and the probability of parasitism. To assess the model's quality, three key statistical terms were calculated: residual deviation, null deviation, and the Akaike Information Criterion (AIC). The residual deviation refers to the sum of squared differences between the observed values and the values predicted by the model. A lower residual deviation indicates that the model fits the data better. The null deviation is the deviation calculated for a model that does not include any explanatory variables, i.e., a model that only predicts the mean of the observed data. It serves as a point of comparison to evaluate the model fit. The Akaike Information Criterion (AIC) is a measure used to assess the quality of a statistical model, considering both the model's goodness of fit and its complexity (number of parameters). A lower AIC value indicates a model that balances both fitting the data well and being parsimonious in terms of the number of parameters. These terms help evaluate whether the proposed model is suitable for explaining the relationship between host body size and the probability of parasitism, considering both the model's fit and complexity.

The model was defined as follows:


$$logi(P(\text{Parasites}))=\cdot \beta_{0}+\cdot \beta_{1}\cdot \times \cdot \text{length}$$ (1)

Where:

*logit* is the logit function, which transforms probabilities into log-odds;

*P*(*Parasites*) is the probability of a frog being parasitized;

𝛽_0_ is the model intercept; and

𝛽_1_ is the coefficient associated with the predictor variable ‘*length*'.

Considering the lack of normality for host body size (W = 0.92497, p = 4.018e-06) and parasitic abundance (W = 0.28249, p < 2.2e-16), as assessed by the Shapiro-Wilk test, we used the Spearman correlation test (*rs*), a non-parametric test, to check for correlations between these variables, with the null hypothesis that there was no significant correlation between host body size and parasitic abundance.

To better understand the parasite-host interaction, we conducted interaction network analyses to visualize the connections between parasites and hosts. Additionally, we examined interaction stronger asymmetry using Blüthgen’s method, which quantifies imbalances in mutual dependence between species within an ecological network. Network structure was assessed through metrics such as the clustering coefficient (a measure of how connected a species' neighbors are; the coefficient ranges from 0 to 1, where values closer to 1 indicate a greater propensity for cluster formation), modularity (Q) (which quantifies the division of the network into distinct groups or communities), and nestedness (the degree to which interactions of species with fewer connections are a subset of those with more connections). We investigated compartment diversity and the presence of clusters and hierarchies within the network organization. Weighted network analyses were performed, including NODF (nestedness metric based on overlap and decreasing fill), weighted nestedness, weighted connectance (the degree of connectivity between species) and specialization among parasites (the degree to which parasites are host-specific).

In ecological network analysis, niche overlap measures the similarity in interaction patterns between species, specifically within bipartite networks. It is calculated using Horn's index, which quantifies the extent to which two species share interactions with the same resources or partners. The formula for Horn's index is:


$$Oij=\;\frac{\Sigma k\;\sqrt{pikpjk}}{\sqrt{\Sigma kp²ik\Sigma kp²jk}}$$ (2)

Where:

*Oij*​ represents the niche overlap between species *i* and *j*;

*pik*​ is the proportion of interactions species *i* has with resource *k*;

*pjk*​ is the proportion of interactions species *j* has with resource *k*.

Values close to 0 indicate no overlap, while values close to 1 indicate perfect overlap, where two species interact with the same resources in the same way. For parasites and hosts, niche overlap refers to the degree of resource or habitat sharing. For parasites, values close to 0 mean different parasites prefer distinct hosts or environments, while values near 1 suggest competition for the same hosts and habitats. For hosts, values close to 0 mean species occupy different niches with minimal competition or shared exposure to parasites, whereas values near 1 indicate that host species interact with the same resources, increasing the likelihood of encountering similar parasitic species.

Network analyses and graph creation were conducted using the software Gephi 0.10.1 (Bastian et al., 2009) and R version 4.3.2 (R Core Team, 2022) through the implementation of the packages bipartite (Dormann et al., 2008), ggplot2 (Wickham, 2016), igraph (Csardi & Nepusz 2006), and vegan (Oksanen et al., 2025). Research data is only available upon request.

## Results

For the parasitological investigation, a total of 276 anurans were sampled (*P. azureus*= 76; *B. raniceps*= 66; *P. platensis*= 61; *S. nasicus*= 58; *S. acuminatus*= 15), 121 of which (43.84%) were parasitized. The parasitism prevalence rates varied among hosts, being 53.94% for *P. azureus* (n = 41), 57.37% for *P. platensis* (n = 35), 45.45% for *B. raniceps* (n = 30), and 33.33% (n = 5) for *S. acuminatus*. The lowest rates were recorded in *S. nasicus* with a prevalence of 17.24% (n = 10).

With a total of 6,165 endoparasite metazoans were collected, 85.94% (n = 5,298) of the specimens were immature forms of Diplostomidae (Trematoda) that had been encysted in organ tissues and the coelomic cavity. In addition to Diplostomidae, other digenetic parasites were identified: *Brachylaima* sp., *Catadiscus cohni* Travassos, 1926, *Catadiscus uruguayensis* Freitas and Lent, 1938, *Choledocystus elegans* Travassos, 1926, *Glypthelmins* cf. *quieta* Stafford, 1905, *Glypthelmins* sp., and *Rauschiella* sp. (Table 1).

**Table 1:** Prevalence, intensity, mean intensity, mean abundance, discrepancy index, infection sites, and host type (intermediate or definitive) of the parasites of hylid anurans from the Nhecolândia Pantanal, State of Mato Grosso do Sul, Brazil.

**Taxa/Parasites**	**Prevalence**	**Intensity**	**Mean intensity**	**Mean abundance**	**Discrepancy Index (d)**	**Infection sites**	**Host species**	**Host type**
Acanthocephala								
Cystacanths	1.51% (1/66)	1	1	0.00 ± 0.12	0.970	Musculature	*Boana raniceps*	Intermediate
								
Trematoda								
*Brachylaima* sp.	1.63% (1/61)	6	6	0.02 ± 0.40	0.968	Musculature	*Pseudis platensis* *	Intermediate
								
Diplostomidae gen. sp.	16.66% (11/66)	3,384 (1 - 1686)	307.63 ± 489.70	44.56 ± 210.45	0.929	Stomach, small and large intestines, coelomic and thoracic cavities	*Boana raniceps*	Intermediate
	28.94% (22/76)	1,102 (1 - 301)	50.09 ±75.66	14.5 ± 46.11	0.885	Urinary bladder, stomach, small and large intestines, liver, coelomic and thoracic cavities, pericardium, and the surface of the lung	*Pithecopus azureus*	
	19.67% (12/61)	483 (1 - 173)	40.25 ±59.80	7.918 ± 30.27	0.923	Stomach, small and large intestines, coelomic and thoracic cavities	*Pseudis platensis*	
	6.89% (4/58)	329 (2 - 232)	82.25 ± 106.60	5.67 ± 32.25	0.955	Stomach, small and large intestines, coelomic and thoracic cavities	*Scinax nasicus*	
								
*Catadiscus cohni*	7.57% (5/66)	6 (1 - 2)	1.02 ± 0.45	0.10 ± 0.35	0.920	Stomach, small and large intestines	Boana raniceps*	Definitive
	5.26% (4/76)	8 (1 - 5)	2 ± 2	0.11 ± 0.60	0.955	Stomach, small and large intestines	Pithecopus azureus*	
	19.67% (12/61)	19 (1 - 4)	1.58 ± 0.90	0.31 ± 0.74	0.839	stomach, small and large intestines	Pseudis platensis*	
								
*Catadiscus uruguayensis*	6.06% (4/66)	5 (1 - 2)	1.25 ± 0.50	0.08 ± 0.32	0.934	Small and large intestines	Boana raniceps*	Definitive
	6.67% (5/76)	13 (1 - 5)	2.5 ± 1.67	0.17 ± 0.76	0.942	Small and large intestines	Pithecopus azureus*	
	22.95% (14/61)	24 (1 - 5)	1.71 ± 1.07	0.39 ± 0.88	0.817	Small and large intestines	Pseudis platensis*	
								
*Choledocystus elegans*	6.55% (4/61)	24 (1 - 13)	6 ±6	0.39 ± 1.96	0.949	Small and large intestines	Pseudis platensis*	Definitive
								
*Glypthelmins* sp.	7.57% (5/66)	96 (1 - 77)	19.2 ± 32.53	1.45 ± 9.56	0.960	Stomach, small and large intestines	*Boana raniceps*	Definitive
								
*Glypthelmins* cf. *quieta*	6.06% (4/66)	58 (1 - 32)	14.5 ± 14.15	0.88 ± 4.63	0.952	Stomach, small and large intestines	Boana raniceps*	Definitive
								
*Rauschiella* sp.	12.12% (8/66)	56 (1 - 32)	7 ± 10.34	0.85 ± 4.10	0.935	Small and large intestines	Boana raniceps*	Definitive
	26.22% (16/61)	45 (1 - 13)	2.81 ± 3.39	0.74 ± 2.10	0.852	Small and large intestines	Pseudis platensis*	
								
Cestoda								
*Cylindrotaenia* sp.	1.63% (1/61)	6	6	0.09 ± 0.77	0.968	Small intestine	Pseudis platensis*	Definitive
								
Nematoda								
Cosmocercidae gen. sp.	4.54% (3/66)	6 (1 - 3)	2 ±1	0.09 ± 0.45	0.950	Small and large intestines	*Boana raniceps*	Definitive
	13.15% (10/76)	148 (1 - 47)	14.8 ± 12.40	1.95 ± 6.54	0.912	Small and large intestines	*Pithecopus azureus*	
	1.72% (1/58)	4	4	0.07 ± 0.53	0.966	Small and large intestines	*Scinax nasicus*	
								
*Aplectana hylambatis*	1.51% (1/66)	6	6	0.09 ± 0.77	0.970	Small and large intestines	*Boana raniceps*	Definitive
	3.94% (3/76)	48 (2 - 43)	16 ± 23.39	0.63 ± 4.70	0.970	Small and large intestines	*Pithecopus azureus*	
								
*Raillietnema minor*	3.94% (3/76)	53 (7 - 38)	17.67 ± 17.62	0.70 ± 4.56	0.963	Small and large intestines	*Pithecopus azureus*	Definitive
								
*Cosmocerca parva*	1.51% (1/66)	2	2	0.03 ± 0.25	0.970	Small and large intestines	*Boana raniceps*	Definitive t
	9.21% (7/76)	26 (1 - 15)	3.71 ±5.12	0.34 ± 1.81	0.946	Small and large intestines	*Pithecopus azureus*	
	6.66% (1/15)	1	1	0.07 ± 0.26	0.875	Small and large intestines	*Scinax acuminatus*	
								
*Cosmocercella minor*	11.84% (9/76)	120 (2 - 52)	13.33 ± 15.67	1.58 ± 6.71	0.931	Small and large intestines	*Pithecopus azureus*	Definitive
								
*Oxyascaris oxyascaris*	10.60% (7/66)	18 (1 - 6)	2.57 ±2.07	0.27 ± 1.02	0.920	Stomach, small and large intestines	*Boana raniceps*	Definitive
	1.31% (1/76)	1	1	0.01 ± 0.11	0.974	Stomach, small and large intestines	*Pithecopus azureus*	
								
Kathlaniidae gen. sp.	9.09% (6/66)	14 (1 - 5)	2.33 ± 2.07	0.23 ± 0.92	0.930	Stomach and small intestine	*Boana raniceps*	Definitive
	6.66% (1/15)	1	1	0.07 ± 0.26	0.875	Small intestine	*Scinax acuminatus*	
								
*Brevimulticaecum* sp.	1.51% (1/66)	10	10	0.15 ± 1.23	0.970	Stomach and small intestine	*Boana raniceps*	Intermediate
	1.63% (1/61)	1	1	0.02 ± 0.13	0.968	Stomach and small intestine	*Pseudis platensis*	
	6.66% (1/15)	1	1	0.07 ± 0.26	0.875	Stomach and small intestine	*Scinax acuminatus*	
	1.72% (1/58)	3	3	0.05 ± 0.39	0.966	Stomach and small intestine	*Scinax nasicus*	
								
*Physaloptera* sp.	3.03% (2/66)	2	1	0.03 ± 1.17	0.985	Stomach and small intestine	*Boana raniceps*	Paratenic
	1.72% (1/58)	18	18	0.31 ± 2.36	0.966	Stomach and small intestine	*Scinax nasicus*	
								
*Rhabdias breviensis*	6.66% (1/15)	1	1	0.07 ± 0.26	0.875	Lung	*Scinax acuminatus*	Definitive
								
Oligochaeta								
*Dero lutzi*	1.51% (1/66)	3	3	0.05 ± 0.37	0.970	Kidneys	*Boana raniceps*	This species follows a life strategy that alternates between free-living periods in aquatic environments and endoparasite stages.
	6.66% (1/15)	2	2	0.13 ± 0.52	0.875	Kidneys	*Scinax acuminatus*	
	3.44% (2/58)	17 (2 - 15)	8.5 ± 9.19	0.29 ± 1.98	0.962	Kidneys	*Scinax nasicus*	

* The first record of the parasite species for these host species, as reported in this study.

The identified Nematoda species accounted for 7.85% (n = 484) of the total number of observed parasites, which included *Aplectana hylambatis* Baylis, 1927, *Cosmocerca parva* Travassos, 1925, *Cosmocercella minor* Steiner, 1924, Cosmocercidae gen. sp., Kathlaniidae gen. sp., *Oxyascaris oxyascaris* Travassos, 1920, *Physaloptera* sp., *Raillietnema minor* Freitas and Dobbin Junior, 1961, and *Rhabdias breviensis* Nascimento et al., 2013. For some larvae, identification at the genus level was possible due to the morphology and positioning of morphological structures, such as the configuration of the ventricle, position of the excretory pore, and excretory nucleus, as observed in *Brevimulticaecum* sp. (Nematoda: Ascarididae). However, in some cases, identification was only possible at the family level due to the absence of males in the samples. Females of different species within certain families can be morphologically very similar to each other, as can their larvae, making species differentiation challenging when relying solely on these developmental stages. Therefore, a more precise taxonomic diagnosis generally requires male morphology, which limits identification when only females or larvae are available.

The less abundant groups were Cestoda, represented by *Cylindrotaenia* sp. (n = 6), and Acanthocephala (n = 1). The latter was encysted and did not allow for further identification at additional taxonomic levels. Additionally, endoparasite Oligochaeta individuals, *Dero lutzi* (n = 22), were identified.

**Figure 1:**
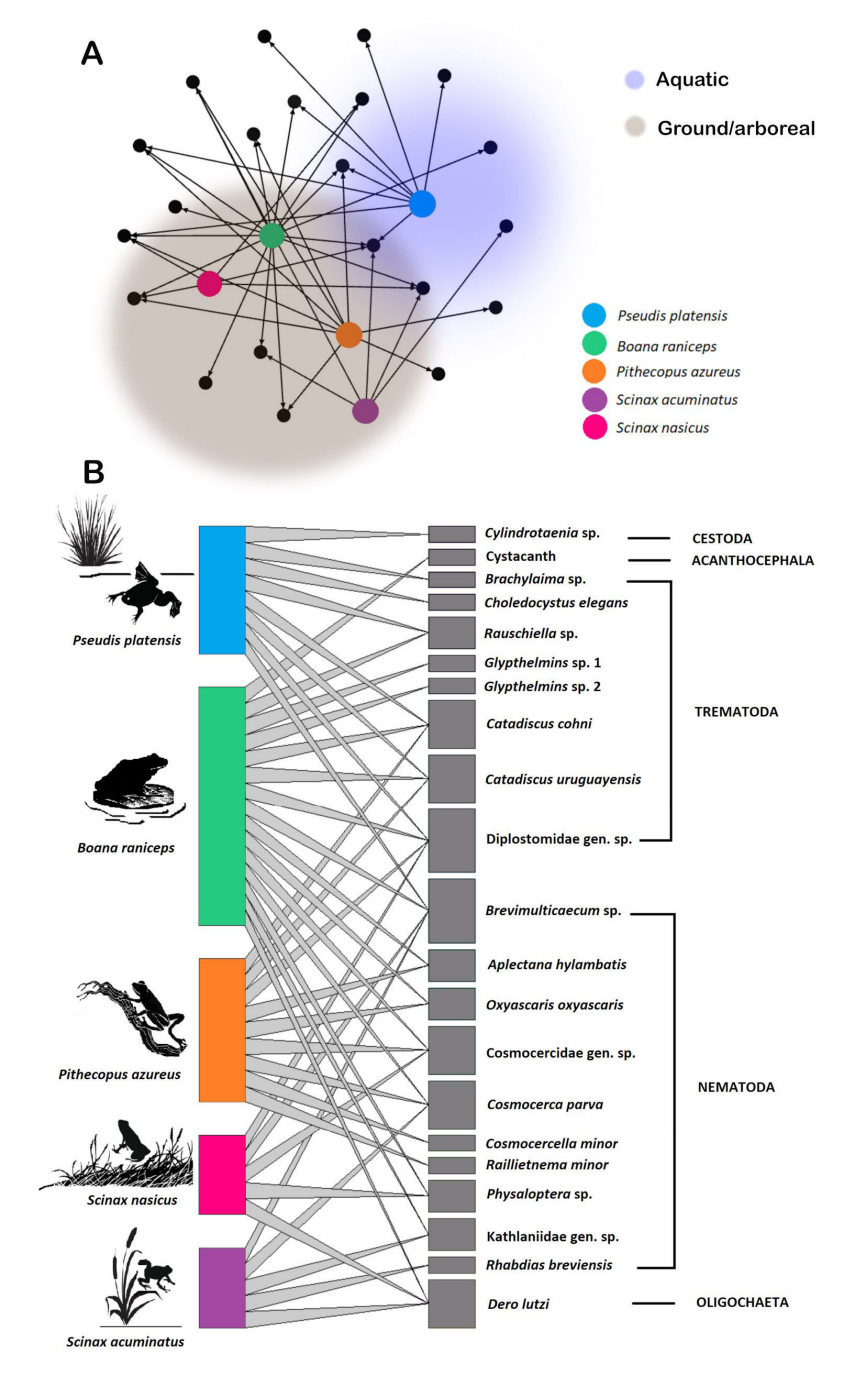
Parasite-host interaction network. A) Network highlighting the connections between hosts and parasites, categorized according to the hosts' habits. The aquatic frog (*Pseudis platensis*) is highlighted in blue, while tree frogs with greater ground exposure (*Boana raniceps*, *Pithecopus azureus*, *Scinax acuminatus* and *Scinax nasicus*) are highlighted in gray. B) Representation of the bipartite parasite-host interaction network structured from a presence-absence matrix. On the left, the host species are illustrated based on their respective habitats. On the right, parasite species are organized according to their taxonomic levels.

*Boana raniceps* stands out as the anuran host species with the highest richness, housing 15 species (Figure 1) and exhibiting a high parasitic abundance, accounting for 3,667 parasite individuals. However, despite the significant richness of parasitic species, the parasitic community presented a low diversity index (H' = 0.419). This scenario is explained by high dominance (D = 0.852), resulting in low equitability (J' = 0.154), where some parasite species, such as Diplostomidae gen. sp., considerably outnumber the others.

In the case of *P. azureus*, the species with the second highest parasitic richness (9 species) and abundance (1,519 individuals), the diversity was relatively high, reaching H' = 1.029. However, this community still exhibited a relatively unequal distribution among species, as indicated by J' = 0.468. The dominance (D = 0.544) reveals a moderate degree of predominance of Diplostomidae gen. sp. over others.

Additionally, when we analyzed the parasites in *P. platensis*, we identified eight distinct species, totaling 608 individuals. The results reveal a high species diversity (H' = 0.840). However, the distribution among species is unequal, indicating a more prominent presence of some species (J' = 0.404), as in the case of Diplostomidae gen. sp., which is corroborated by the dominance (D = 0.640). On the other hand, *S. nasicus* was found to harbor five species of parasites, totaling 371 individuals. Although the diversity (H') is moderate, reaching 0.482, the distribution among species is relatively unequal (J'= 0.299), suggesting considerable dominance (D = 0.791) of Diplostomidae gen. sp. in the community and highlighting a moderate degree of dominance.

### Parasitism and host body size

Average body size (SVL) of hosts was 58.76 ± 3.25 mm (48.99-64.34 mm) for *B. raniceps*, 40.77 ± 3.08 mm (35.82-47.36 mm) for *S. acuminatus*, 39.89 ± 4.12 mm (30.75-50.36 mm) for *P. platensis*, 34.17 ± 5.22 mm (20.96-47.00 mm) for *P. azureus*, and 30.11 ± 3.13 mm (23.03-36.91 mm) for *S. nasicus*.

The results revealed that the intercept (𝛽_0_ = −0.86986) and the coefficient for the host body size (𝛽_1_ = 0.01677) were not statistically significant (p > 0.05), indicating that, in the absence of other variables, the probability of an anuran being parasitized may not be different from zero and that the host body size may not be a significant predictor of the probability of parasitism. Furthermore, the residual deviation was 369.54 with 268 degrees of freedom, whereas the null deviation was 371.79 with 269 degrees of freedom. The Akaike information criterion (AIC) was 373.54, providing a metric for assessing the model's quality. These results indicate that, based on the fitted model, the host body size does not have a significant influence on the probability of being parasitized. The Spearman correlation test indicated that the correlation coefficient between the variables “length” and “parasitic abundance” was equal to -0.1803 and the p < 0.05 (*rs* = 0.0468), suggesting that the observed correlation was statistically significant; however, this negative correlation was weak.

### Analysis of interaction networks

Network analysis at the general level revealed a connectance of 40%, demonstrating a significant number of interactions (Figure 1). The value of 0.615 for network asymmetry (ranging from 0 to 1, where values closer to 1 indicate strong asymmetry in interactions) suggests that some parasite species interact with a greater number of host species than the reverse. In other words, certain hosts are associated with fewer parasite species (Figure 1). With an average of 1.62 links per species, we observed moderate connectivity among host and parasite species in the network.

The linkage density was calculated at 6.26 links per unit, indicating that each species interacts with approximately six other species in the network. For the assessment of niche overlap, we observed two values, one for hosts (approximately 0.40) and one for parasites (approximately 0.36). Considering that niche overlap measures the extent to which species share resources or occupy similar niches, the provided values indicate that there is moderate niche overlap in both host and parasite interactions.

The clustering coefficient was 0.4, suggesting a relatively modular organization in the network, with local clusters of interactions. The modularity (Q) was 33%, indicating a relatively modular structure, with groups of species more interconnected with each other than with other species outside the group. The nestedness reached 31.35%, suggesting a nonrandom pattern of interactions, where less connected species interact with a subset of more connected species (Figure 1).

The NODF (nestedness overlap and decreasing fill), which measures the degree of nesting in an ecological network, presented a value of 49.90%, indicating a nested structure; the weighted nestedness, reflecting the intensity of this nesting, was 0.29. The value of the interaction strength asymmetry is 0. This suggests that, based on the interactions in the analyzed network, there is no asymmetry in interaction strength among the species. In other words, the strengths of the interactions are equitable and do not significantly differ in direction or magnitude between species. The specialization asymmetry value is approximately -0.385, indicating that asymmetric specialization may suggest that some species are more specialized in their interactions than others are. Additionally, the negative value may suggest that there is asymmetry in how species specialize in their interactions.

## Discussion

We report a significant prevalence of parasites, infecting nearly half of the hylid anurans studied in the Nhecolândia Pantanal region, and provide novel records of these parasite species, expanding their known host range to include *Brachylaima* sp. in *P. platensis*, *Catadiscus cohni* and *Catadiscus uruguayensis* in *B. raniceps*, *P. azureus*, and *P. platensis*, *Choledocystus elegans* in *P. platensis*, *Glypthelmins* cf. *quieta* in *B. raniceps*, *Rauschiella* sp. in *B. raniceps* and *P. platensis*, and *Cylindrotaenia* sp. in *P. platensis* (Table 1).

Furthermore, we observed the predominance of metacercariae from Diplostomidae gen. sp., accounting for 85.94% of the total, with an aggregated distribution across all host species where they were present (*B. raniceps*, *P. azureus*, *P. platensis* and *S. nasicus*). According to the general quantitative results of the infracommunities of endoparasite metazoans (Table 1), immature forms of Diplostomidae gen. sp. presented the highest prevalence for each examined host species. This pattern was also observed for other metrics, such as intensity, mean intensity, and mean abundance, demonstrating the typical aggregated distribution pattern.

Since the aggregated distribution pattern remains predominant in most host-parasite systems for metazoans, this can be explained by exposure to infective stages in the shared environment of the hosts, followed by their capacity to resist infection (immunity) (Warburton & Vonhof, 2018). The use of microhabitats, such as the proximity of these anurans to water, potentially facilitates the infection process for trematodes, as this environment is directly linked to both the lifestyle of these hylids and the life cycle of the Trematoda (Bray et al., 2008). In the present study, we identified parasites such as *Brachylaima* sp. in *P. platensis*, where the adult forms are found in the digestive tracts of birds and mammals (Butcher & Grove, 2001; Taroda et al., 2013), with a mollusk serving as the first intermediate host, whereas amphibians and lizards may act as the second intermediate host (Thiengo & Amato, 1995; Aisien et al., 2017; Fedatto-Bernardon et al., 2017). Adult trematodes, such as *Catadiscus cohni*, *Catadiscus uruguayensis*, *Choledocystus elegans*, *Glypthelmins* sp.1, *Glypthelmins* sp.2 and *Rauschiella* sp., which are commonly found in anurans (Fernandes & Kohn, 2014; Campião et al., 2014) and involve gastropods as intermediate hosts (Rankin, 1944; Martin, 1969; Yamaguti, 1975; Kehr & Hamann, 2003; Hamann, 2006), were also observed.

In addition to parasites with complex life cycles involving one or two intermediate hosts, parasites with simplified life cycles have been reported, such as the Cestoda *Cylindrotaenia* sp. The first report in Brazil of experimental studies on the life cycle in tadpoles and terrestrial adults of *Rhinella icterica* (Spix, 1824) concluded that the infection of this parasite has a direct cycle (Stumpf, 1981, 1982). In the present study, *Cylindrotaenia* sp. was observed only in *P. platensis*, a fact that may be related to the aquatic lifestyle of this anuran species (Duellman & Trueb, 1986), potentially facilitating infection.

Life cycles without the need for intermediate hosts are found in nematodes, such as specimens from the family Cosmocercidae, which are common intestinal parasites in amphibians and reptiles (Vicente et al., 1990; Ramallo et al., 2008; Campião et al., 2014; Silva et al., 2024a). These nematodes can be transmitted through the active penetration of larvae into the host's skin or through the ingestion of infective larval forms (Anderson, 2000), as exemplified by members of the genera *Raillietnema* (Moravec & Řehulka, 1987), *Cosmocerca* (González & Hamann, 2012), and *Aplectana* (Anderson, 2000). Experimental studies have demonstrated that juvenile forms of *Aplectana courdurieri* in aquatic environments are unable to infect *Rana mascareniensis* (= *Ptychadena mascareniensis* (Duméril & Bibron, 1841)) via skin penetration, suggesting that species of *Aplectana* may have terrestrial life cycles, similar to those of other cosmocercids (Anderson, 2000).

Some nematodes, such as members of the family Kathlaniidae, may involve more actors in their life cycle (Anderson, 2000). The presence of third-stage larvae of *Falcaustra* spp. has been reported in fish (Moravec et al., 1995) and mollusks, suggesting that they are paratenic hosts (Bartlett & Anderson, 1985). Adult forms are observed more frequently in chelonians than in amphibians, fish, and birds (Baker, 1987), but in our study, adult females were observed in *B. raniceps* and *S. acuminatus*. On the other hand, while species of *Brevimulticaecum* typically use aquatic reptiles (especially crocodilians) and rays as definitive hosts (Goldberg et al., 1991; Rego, 1978; Sprent, 1979; Moravec et al., 1997; Reyda, 2008), the occurrence of larval stages of *Brevimulticaecum* sp. was recorded in all studied anuran species except *P. azureus*, suggesting that *B. raniceps*, *P. platensis*, *S. acuminatus* and *S. nasicus* may play a role as intermediate or paratenic hosts (Moravec & Kaiser, 1994).

Other nematodes with heteroxenous life cycles belong to the family Physalopteridae, which consists primarily of parasites found in the stomachs of reptiles, birds, mammals, amphibians, and fish, where they are usually found firmly attached to the gastric mucosa with the aid of large, dentate pseudolabia (Anderson, 2000). Intermediate hosts include invertebrates such as the cockroach *Blattella germanica* (Linnaeus, 1767) (Roth & Willis, 1957) and vertebrates such as amphibians and snakes may serve as paratenic hosts (Anderson, 2000). In this study, the observed stages in the anurans were larval stage, leading to the conclusion that the anuran species parasitized by these metazoans, *B. raniceps* and *S. nasicus*, are paratenic hosts that likely become infected through trophic means; despite having a generalist diet, insects are the main food items of these hylids (Sabagh et al., 2010; Cossovich et al., 2011).

In turn, the oligochaete *Dero lutzi* Michaelsen 1926, which has a life strategy that alternates between free-living stage in aquatic environments and endoparasite stage on urinary system of anurans (Lutz, 2007; Silva et al., 2024b), was found to parasitize the urinary tracts of *B. raniceps*, *S. acuminatus*, and *S. nasicus* in the present study. The record of *D. lutzi* in *Scinax fuscovarius* and *S. nasicus* in the state of Mato Grosso do Sul, including our study area, was recently reported (Silva et al., 2024b); these authors report that *D. lutzi* shares ancestry with the free-living oligochaete *Dero superterrenus* and suggest that the free-living ancestor of these species (*D. lutzi* and *D. superterrenus*) may have exhibited parasitic and/or phoretic behaviors while exploring anurans.

*Scinax* (*S. acuminatus* and *S. nasicus*) presented the lowest values of abundance and richness of endoparasites (Table 1; Figure 1). Additionally, *S. acuminatus* was the species with the lowest abundance in the sampled area, which can be attributed to its frequent association with habitats adjacent to wetlands and terrestrial bromeliads as refuges (Duré & Schaefer, 2011; Caballero-Gini et al., 2021). These hylids are primarily arboreal and use the axillary leaves of bromeliads, herbaceous vegetation, shrubs, and even human dwellings as microhabitats (Ávila & Ferreira, 2004; Sabagh et al., 2010; Duré & Schaefer, 2011). Their diet, which is predominantly composed of spiders and insects from the orders Diptera, Orthoptera, Blattodea, Hymenoptera (e.g., ants), and Coleoptera (e.g., Scarabaeidae) (Duré, 1999; Sabagh et al., 2010; Cossovich et al., 2011; Thaler et al., 2021), may explain the sharing and association of these species with the ground, thereby partly justifying the low richness of their endoparasite assemblages. Because their diet includes a high proportion of terrestrial arthropods, they may have reduced exposure to certain parasite taxa that rely on aquatic transmission routes. Consequently, their limited interaction with aquatic habitats likely restricts their exposure to a broader diversity of endoparasites, resulting in a lower richness of parasites in these species.

The body size of the anurans in the study was not a strong predictor of parasitism rates. Furthermore, this same variable and parasitic abundance were weakly negatively correlated. Although this association was significant, the level of significance was very close to the threshold (*rs*= 0.0468), and it does not seem to be a deterministic pattern, as the specific characteristics of each investigated species are more expressive. Notably, *B. raniceps* presented the greatest average length (58.76 ± 3.25 mm) among the anurans, which was proportional to the abundance of parasites (3,658). For the other species, we did not observe the same pattern, corroborating the findings of Ward et al. (2002), who reported no relationship between host body size and the abundance or prevalence of parasites in fish, suggesting that host body size and parasitic status are independent predictors.

Parasites are often generalists, but they are associated with specific types of functional groups or dietary guilds of their hosts. By sharing lifestyles and dietary preferences, members of these guilds often exhibit similarities in their parasitic fauna, whose constituent species follow common transmission pathways (Marcogliese, 2002; Campião et al., 2015). The results obtained from the analysis of parasitic networks among hylid anurans and their parasites contribute to a better understanding of the dynamics of these interactions in the studied ecosystem. The network demonstrated a significant interaction between parasites and host species, with moderate connectivity among species in the network. Furthermore, the asymmetry in the network structure suggests that some species may have more direct interactions with others than the reverse, indicating that certain species of endoparasites may exert a more intense influence on the network than others do.

Most parasites that are transmitted trophically are associated with a specific niche and diet of the host (Marcogliese, 2002). The order Anura is characterized by encompassing both opportunistic predators and specialized predators (Toledo et al., 2007). Our results indicate that there is moderate niche overlap among the interactions, meaning that at some point in the natural history of these anuran species, the adults share the same microhabitat. Reproductive activities may involve site selection strategies for vocalization (e.g., Delatorre et al., 2015; Martins et al., 2021), egg laying (and tadpole development), and foraging, which can occur in sympatry (e.g., Sabagh et al., 2010; Delatorre et al., 2015; Martins et al., 2015). In this sense, the results were more pronounced for metacercariae, which corroborates the findings of field experiments that parasites can increase energy flow rates along certain trophic links.

That is, parasitized intermediate hosts may be more susceptible to predation by definitive hosts (Lafferty & Morris, 1996; Thomas & Poulin, 1998). This can be interpreted as a way for the parasite to increase the likelihood of predation by the final host (e.g., birds) (Rohde, 2005). An example is the predation of *B. raniceps*, *P. azureus*, *P. platensis* and *S. nasicus* by birds in natural environments (Toledo et al., 2005; Landgref Filho et al., 2011 ; Landgref Filho et al., 2019). The results indicate a modular structure in the interaction network between helminths and anurans. This modular structure suggests the formation of specific communities of helminths and anurans, each with its own ecological dynamics, which may reflect evolutionary adaptations, as it is possible to observe some links more characteristic of the anuran habitat (such as trematodes). In a broader context, the network reveals that, at some point in the adult life of these amphibians, the species share the same microhabitat, as evidenced by the presence of certain parasites in the same anuran species. A notable example of this phenomenon is the high incidence of hosts parasitized by trematodes. The strong associations between anurans and microhabitats in and around bodies of water are directly linked to the reproductive modes and intrinsic attributes of amphibians (Prado et al., 2005; Caballero-Gini et al., 2021). The selection of these microhabitats is influenced by the reproductive mode of the group, which is affected by physical parameters, especially hydroperiod and volume, as well as the chemical properties of the water bodies used for reproduction, in association with the structural complexity of the surrounding vegetation (Oliveira & Eterovick, 2010 ; Delatorre et al., 2015).

These elements play crucial roles in reproductive biology, highlighting the interaction between the environment and the reproductive strategies of anuran species. The overlap of the reproductive periods of the species sampled in this study-*B. raniceps*, *P. azureus*, *P. platensis*, *S. acuminatus* and *S. nasicus*—corroborates the periods reported by Prado et al. (2005). Given these results and assuming that the organization of ecological networks is influenced by biological attributes, such as habitat heterogeneity (Marcogliese, 2002; Schleuning et al., 2012; Flores et al., 2013), and that the considered species exhibit differentiated uses of microhabitats (Haddad et al., 2013), it can be concluded that these species are subjected to different exposures to parasitic agents. Thus, the distinct use of the environment by the host species exposes them to potential infections according to the biological cycles of the parasites, positioning the host communities as determinants for aspects of parasitic communities (Lafferty, 2008).

In conclusion, this study provides a comprehensive understanding of the complex interactions between helminths and anurans, highlighting the importance of considering not only the presence but also the intensity of these interactions in the ecology of parasitic networks. This approach to specific ecological dynamics contributes to research in community ecology, not only of the host species but also of their respective parasites.
